# Dissecting the dimension of protection: *Caligae* and *Scutum* in the evaluative model of normative appeals

**DOI:** 10.1007/s12144-021-02209-1

**Published:** 2021-08-21

**Authors:** Luis Oceja, Maite Beramendi, Sergio Salgado, Pablo Gavilán, Marisol Villegas

**Affiliations:** 1 Departamento de Psicología Social, Universidad Autónoma de Madrid, C/ Pavlov 6, 28049 Madrid, Spain; 2 Universidad de Buenos Aires-CONICET, Buenos Aires, Argentina; 3 Departamento de Administración y Economía, Universidad de La Frontera, Temuco, Chile; 4 Centro de Estudios del Desarrollo, Universidad Central de Venezuela, Caracas, Venezuela

**Keywords:** Appraisal, Compliance, Formality, Norms, Protection

## Abstract

A normative appeal indicates that one should (or should not) do a certain action in a concrete situation. According to the Evaluative Model of Normative Appeals (EMNA), willingness to comply with these messages depends on an appraisal formed by two dimensions: formality and protection. In this work we center on the dimension of protection, proposing that it can be divided into two components: avoiding physical or psychological damage (*scutum*) and affording the performance of the main intended action (*caligae*). We conducted two studies to test this twofold meaning of protection. In Study 1 (*N* = 525), we manipulated the coherence of regulatory focus (promotion vs. control vs. prevention) with salience of the components of protection (*caligae* vs. control vs. *scutum*). In Study 2 (*N* = 513), we separately measured the perception of each component referred to an actual normative appeal (i.e., “To get into a class punctually”). The results showed that the manipulated salience and the measured perception of *caligae* and *scutum* elicits (Study 1) and predicts (Study 2) higher willingness to comply with normative appeals. Theoretical and applied implications of the results are discussed.

*Remember Rilke’s admonition: Love consists in leaving the loved one space to be themselves while providing the security within which the self may flourish.*

The Memory Chalet (Judt, 2010, p. 66).

According to the Evaluative Model of Normative Appeals (EMNA), willingness to comply with a normative appeal is closely related to what, in the Rilke (1875–1926) admonition above, is the essential aspect of love: the ability to create a safe space that allows moving toward the intended path. Judt (2010) uses this quote to express the happiness he felt whenever he went somewhere on his own; by walking, cycling, travelling in a bus or, especially, on a train. Train journeys were heaven for Judt because they allowed him to be immersed in the awareness of freely and safely moving ahead. We show in this work how this Judt’s explanation of his love for train journeys is used as an analogy to illustrate one dimension of the normative appraisal proposed by the EMNA; that is, a process of perception that exerts influence on the willingness to comply with a normative appeal.

A normative appeal is a message indicating that one should or should not perform a certain action in a situation (e.g., a sign or a phrase requesting silence in a public place). The potential of these messages to direct one’s actions depends on many factors highlighted by previous theoretical approaches. First, the Focus Theory of Normative Behavior (Cialdini et al., 1990; Cialdini et al., 1991; Cialdini et al., 2006; Kallgren et al., 2000) emphasizes the extent to which the message (a) draws one’s attention, and (b) refers to our perceptions and beliefs about what most people do in a given situation (descriptive norm), and about what the reference group considers appropriate to perform (injunctive norm). Second, the Theory of Normative Activation underlines the degree of agreement between personal values ​​activated at the time and the values ​​expressed by the message (Schwartz, 1977). Third, the theories of Reasoned Action (Fishbein & Ajzen, 1975) and Planned Behavior (Ajzen, 1991) highlight the attitude, the perceived control and the opinions of significant others concerning the action proposed by the message. Fourth, the Relational Model of Authority (Tyler, 1997; Tyler & Lind, 1992) and the Group Engagement Model (Tyler & Blader, 2003) stress the importance of the perception of the legitimacy of and identification with the entity that promulgates the message. Fifth, the Deterrence Theory emphasize the utility calculation process through which we estimate the material and social costs and benefits derived from following or passing over what is stated in the message (Gibbs, 1968).

Likewise, in their Evaluative Model of Normative Appeals (EMNA), Oceja et al. (2016) propose to add a new factor to this list: a two-dimensional process (i.e., *normative appraisal*) through which the person evaluates the extent to which a normative appeal is formal and protective. The dimension of formality refers to whether the appeal comes from a relatively informal source (e.g., family, friends, and acquaintances) or a formal institution (e.g., the university, council, or government). The dimension of protection refers to the degree to which the normative stating (e.g., you should) of the proposed conduct (e.g., remain silent) is perceived as a factor that prevents relevant damages and/or fosters the performance of the main intended action (e.g., to study).

Regarding the output of this *normative appraisal*, the EMNA has two fundamental assumptions. The first assumption is that the appeal will be then perceived as being relatively formal and protective, and this perception can be conceived as occupying a specific point in a two-dimensional space. The four main areas circumscribed by this space are custom (low formality and low protection), coercive norm (high formality and low protection), prescription (low formality and high protection), and legitimate norm (high formality and high protection) (Oceja et al., 2016). Note that these labels are arbitrary terms used by the researchers to denote two aspects. First, different normative appeals can be perceived in a similar fashion (e.g., “You should not litter” and “You should donate blood” are typically perceived as a prescription). Second, the same normative appeal can be perceived differently depending upon the person-situation combination (e.g., “You should remain silent” can be perceived as a custom, a coercive norm, a prescription, or a legitimate norm).

The second assumption of EMNA is that willingness to comply with a specific normative appeal will depend on the output of this two-dimensional normative appraisal; following a continuum that in ascending order goes from custom to coercive norm to prescription to legitimate norm. Previous research centered on testing those two assumptions (Oceja et al., 2016; Salgado et al., 2018), we now will center on proposing and examining a more refined definition of the dimension of protection.

## Proposing the Twofold Meaning of Protection

In the present work, we add and test a new hypothesis related to the structure of the normative appraisal proposed by the EMNA. Specifically, we state that the dimension of protection should be divided into two components referring to the degree to which the appeal is perceived as (a) avoiding hazards, physical injury, or psychological damage (*scutum*) and as (b) affording the performance of the main current action as intended (*caligae).* This division of protection into two components mainly relies on the Bowlby and Ainsworth’s Attachment Theory (Bretherton, 1992), which defends the existence of an innate system of attachment covering two basic human needs: (a) maintaining a feeling of psychological security that allows (b) exploring autonomously how to achieve relevant objectives (Sroufe & Waters, 1977). In this vein, with respect to these two basic needs, the EMNA proposes that a normative appeal will be evaluated as protective along as it is perceived as providing the feeling of being safe in a psychological and physical sense (*scutum*), and/or of being able to explore for and move toward the performance of the main intended action (*caligae*).

To avoid the misunderstanding usually provoked by the many connotations of terms such as security, safety, freedom, motion and so on, we coined the terms *scutum* and *caligae* (in Latin, the shield and sandals carried by the Roman legionaries) to refer to these two meanings of protection. These qualities of *scutum* and *caligae* are not mutually exclusive or opposite. A normative appeal can be perceived as protective in either one of the two meanings or both—as a train can be perceived as a safe mode of transport, a means to travel wherever one wants, or both. Therefore, we propose that both components should be distinguished and separately measured.

In synthesis, the EMNA centers on the nature and consequences of a specific type of appraisal: the perception of a message that explicitly states how one should (not) in a situation. Previous research suggests that people report greater willingness to comply with normative appeals that are consistently perceived as more protective (Oceja et al., 2016). In this work, we additionally propose that this protection refers mainly to the degree to which the normative appeal is perceived as avoiding damage (*scutum*) and affording the performance of the main intended action (*caligae*). We therefore expect that (a) the activation of *caligae* or *scutum* increase the willingness to comply with those normative appeals usually perceived as protective, and (b) the measurements of perception of *caligae* and *scutum* separately predict the willingness to comply with a specific normative appeal.

## Testing the Twofold Meaning of Protection

Regarding the first expectation, a typical way of proving that a product may have two different functions (e.g., a knife as a weapon or as a cutlery) is by testing whether contextually priming one of these functions increases the perceived utility of the product (Shen & Chen, 2007; Yi, 1993). For example, previously asking people to think about their physical security or food preferences may subsequently increase the value of a knife. However, the effectiveness of a prime depends on its motivational relevance (Higgins & Eitam, 2014; Molden, 2014; Scholer et al., 2019). That is, representations elicited by a prime only become activated for potential use in impressions and behaviors when they are congruent with one’s current motivations (see also Lee & Aaker, 2004). Likewise, according to the Regulatory Focus Theory (Higgins, 1997), a person may be focused on either promoting fulfillment of aspirations and achievements (promotion) or preventing deviations from one’s own duty and liability (prevention). This promotional or preventive focus is activated depending on current personal needs and the situational context. Based on this theory, Higgins and collaborators found the predicted effects on feelings, thoughts, and behavior by consistently manipulating the self-regulatory focus. They have used an ample repertoire of either explicit instructions (e.g., focusing on possible gains vs. losses when buying a product) or indirect priming tasks (e.g., solving a maze in which a mouse tries to get a piece of cheese vs. to escape from an eagle) (for a review, see Higgins, 2012, chapter 8).

We grounded on the positive effect of the focus-prime fit given by Higgins (2012) to address our first expectation concerning the twofold meaning of protection. Specifically, we state two hypotheses by combining this twofold character with the focus-prime fit principle. First, we expect greater willingness when the *scutum* prime is combined with the prevention focus (avoiding hazards), and when the *caligae* prime is combined with the promotion focus (pursuing achievements). Second, this expected effect of the combination of priming and focus will be found mainly for those normative appeals that are perceived as protective (according to the EMNA typology, legitimate norms and prescriptions).

Regarding the second expectation, Oceja et al. (2016) proposes that the willingness to comply with a normative appeal could be predicted –among other processes– by the degree to which the appeal is perceived as protective. Consequently, as we propose that this perceived protection comprises two meanings, we should test whether perceived *caligae* and *scutum* separately predicts actual willingness to comply. Therefore, we selected a context in which a specific normative appeal is being applied (i.e., “You should get into class punctually”) and measured the perception of the two meanings of protection and the willingness to comply with that normative appeal. Our hypothesis is that perceived *scutum* and *caligae* will predict the willingness to comply independently one from each other.

Summing up, we conducted two studies to test the twofold meaning of protection. First, in line with the EMNA and Self-regulatory Theory, we conducted Study 1 to test whether the focus-prime fit of prevention-*scutum* and promotion-*caligae* increases willingness to comply with a (protective) normative appeal. Second, we conducted Study 2 to test whether *scutum* and *caligae* separately predicts the willingness to comply with an actually present normative appeal. The data corresponding to all the variables included in these two studies are available on Open Science Framework (OSF) website (https://osf.io/v5mtx/).

## Study 1: Bringing the Prime into Focus

In Study 1, we examined the relationship between the two regulatory foci (promotion and prevention) and the prime of each of the two meanings of the protection dimension (*caligae* and *scutum*). Specifically, according to the EMNA, perceived protection of a normative appeal may refer to the extent to which the appeal is perceived as providing *caligae* (i.e., allowing one to perform the main action as intended), *scutum* (i.e., avoiding one from suffering physical or psychological damage) or both. Therefore, we reasoned that the dynamic between focus and prime thoroughly supported by previous research (Higgins, 2012) can be applied to further test the twofold meaning of protection. We therefore expected that the prime of each meaning would especially increase the willingness to comply (WTC hereafter) under two conditions: (a) when the prime is motivational relevant (i.e., when there is focus-prime coherence), and (b) when the normative appeal is perceived as protective. We used a 3 (regulatory focus: promotion vs. control vs. prevention) × 3 (prime: *caligae* vs. control vs. *scutum*) between-subjects design.

### Method

#### Participants

A priori power analysis conducted by using G*Power 3.1 (Faul et al., 2009) suggested a minimum sample of 304 to localize main and interaction effects with a medium size effect (*d* = 0.20) in a 3 × 3 between-subjects design with a power of 80%. The sample was collected in two waves in two countries, and it includes 525 participants (255 Spaniards and 270 Argentineans; *M*_age_ = 23.51, *SD*_age_ = 6.12) that were randomly assigned to each of the nine experimental conditions corresponding to the 3 × 3 factorial design.

#### Procedure

We followed the double-blindness procedure in the collection and analysis of the data. First, a researcher approached participants who were seated alone in one of the libraries of the campus. Participants were invited to complete a questionnaire and read a consent form. The researcher also informed them that after the questionnaire they would receive a paper explaining the logic and objective of the study. Approximately 80% accepted, and the researcher gave them a questionnaire with three stapled pages. There were nine versions (3 × 3 factorial design). The first page presented the manipulation of the regulatory focus through one of the techniques developed by Higgins and collaborators (for a review, see Higgins, 2012). Specifically, the participants could read one of three instructions. For the control condition: *Please think about the first thing you now have in mind regarding ideas or feelings. Could you describe it now in short sentences?* For the promotion-focus condition: *Please think about something that you would ideally want to do regarding a wish or an aspiration that you currently have. Could you list now, in short sentences, three of your wishes and aspirations?* For the prevention-focus condition: *Please think about something that you believe you should do regarding a responsibility or an obligation that you currently have. Could you list now, in short sentences, three of your responsibilities and obligations?*

The second page presented one of the three versions of the prime manipulation. For the control condition: *Asking questions to collect information is important and necessary. Please turn this page and answer the presented questions*. For the caligae-prime and scutum-prime conditions: [*Freedom*, *Security*] *is important to everybody, but each person has her/his idea of what it means. Please write four or five aspects that you consider may threaten your* [*Freedom, Security*]. The only difference is in brackets.

The third page included a questionnaire that presented a list of 28 different normative appeals (Oceja et al., 2016) (Table 2). In each country, the researchers printed out these 3-page questionnaires, randomly shuffled them and, being blinded to the version handed out, distributed all of them in two sessions. All the participants who accepted the invitation fully and correctly completed the questionnaire.

##### Hypothesis

We asked participants to indicate to what degree they agreed with each normative appeal on a 9-point scale (1 = not at all, 9 = totally). Our main hypothesis is that the focus-prime congruency (i.e., promotion-*caligae* and prevention-*scutum*) will significantly increase the WCT especially for those normative appeals typically perceived as protective. Additionally, we will test whether the focus (i.e., promotion and prevention) and the prime (caligae and scutum) are sufficient to provoke such increase.

### Results and Discussion

To form the high-protective and low-protective sets of normative appeals, we based on Oceja and collaborators (2016) that allowed us to differentiate between the 16 normative appeals that are typically perceived as a legitimate norm or prescription, and the 12 that are typically perceived as a coercive norm or custom. We therefore combined the WTC reported for these 16 and 12 appeals into two general measures (*α*s = .78 and .71, respectively) that did not depend on the characteristics of a specific appeal; instead, they tapped the WTC elicited by appeals typically perceived as high vs. low protective. The Table 1 presents these 28 appeals along with the weight values obtained after conducting an exploratory factorial analysis (2-factor solution, maximum likelihood, and oblimin rotation). The 16 and 12 high and low protective appeals obtained the highest weight values in the first and second factor, respectively; with the only exception of the not double parking which obtained a moderately high weight value in both factors.

**Table 1:** *Normative appeals presented in Study 1*(All started with “You should”) along with the weight values obtained in a factorial analysis (2-factor solution, maximum likelihood, and oblimin rotation)

1. not illegally download music or movies from the Internet.		.52
2. turn off the lights when you leave^*^.	.30	
3. recycle materials such as paper, glass, plastic, etc.^*^.	.27	
4. not kiss intensely in public places.		.50
5. not speed on the freeway^*^.	.46	
6. stop for pedestrians in the crosswalk^*^.	.49	
7. not leave the water turned on^*^.	.47	
8. not frequently turn your head from side to side during a conversation.		.28
9. not double-park.	.42	.41
10. not smoke in the subway^*^.	.58	
11. not litter^*^.	.67	
12. not block others while looking at a painting in a museum.		.26
13. not fake being sick to skip work^*^.	.42	.29
14. not block the way of an ambulance that has its emergency lights on^*^.	.57	
15. not use sprays that are harmful to the environment^*^.	.45	
16. not eat at the bus stop.		.44
17. not sell pirated copies of CDs.		.50
18. not drive when your blood alcohol level is above the legal limit^*^.	.59	
19. not yell at someone to greet them from a distance.		.50
20. not consume alcohol in public spaces.	.35	.53
21. not damage public property (garbage cans, walls, etc.)^*^.	.65	
22. not ask the person who is going to receive the tip for change.		.29
23. not photocopy books.		.55
24. not sell illegal drugs^*^.	.48	.38
25. donate blood^*^.	.28	
26. not park in a restricted area^*^.	.56	.27
27. not cut in line^*^.	.45	
28. not talk on your cell phone in an elevator or public transportation.		.36

Note: Those with and without an asterisk were included in the high- and low-protective group, respectively. Weight values lower than .25 are not shown

#### Gender and Country

We initially conducted four 2 (gender) × 2 (country) ANOVAs to test whether gender or country significantly interacted with focus or prime on the effect on WTC with high or low protective normative appeals. First, the ANOVAs revealed that WTC with high-protective appeals was higher among women than men [*M*s = 7.95 vs. 7.67, *SD*s = 0.80 and 0.92, respectively, *F*(1, 521) = 9.71, *η*_p_^2^ = .018, *p* = .002], and not any other significant main effects, *F*s < 3.83, *p*s > .07, *η*_p_^2^ < .007. Second, the results did not show any interaction effect, *F*s < 1.97, *p*s > .139, *η*_p_^2^ < .01. Therefore, gender and country did not moderate the influence of focus and prime on WTC and are not discussed further in this research.

#### Focus and Prime

**Fig. 1:**
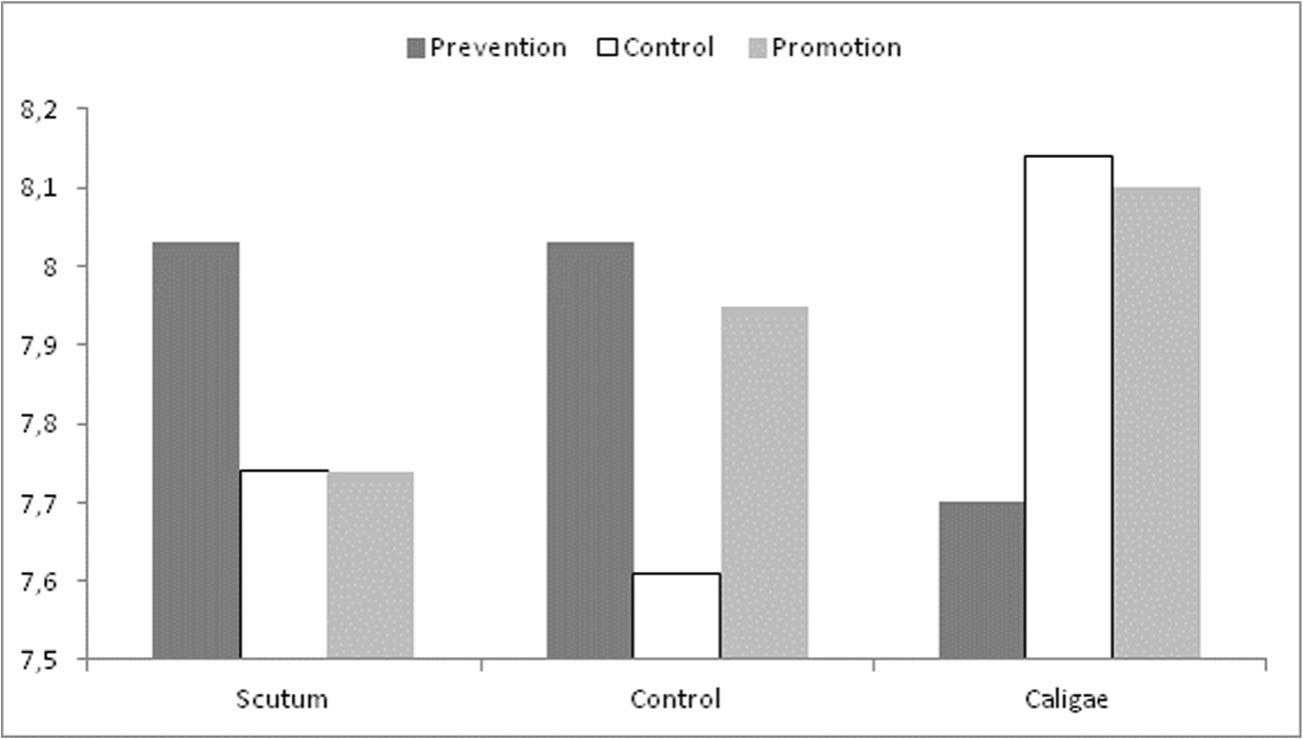
Willingness to comply with the protective normative appeals as a function of regulatory focus and prime

We performed a 3 (focus) × 3 (prime) ANOVA on the general WTC with high protective appeals. The analysis revealed that the main effects of focus and prime were not significant, *F*s(2, 516) < 1.49, *η*_p_^2^ < .01; but the interaction between them was, *F*(4, 516) = 5.40, *η*_p_^2^ = .040, *p* < .001. As can be seen in Fig. 1, this interaction effect was in line with our main hypothesis. Namely, Waller-Duncan post hoc tests (*p* < .05) indicated that the two conditions with a high focus-prime congruency (i.e., promotion-*caligae* and prevention-*scutum*) provoked a higher WTC (*M*s = 8.10 and 8.03, *SD*s = 0.63 and 0.70, respectively) than that provoked in the control (*M* = 7.61, *SD* = 1.02); *d*s = .58 and .48, respectively. In contrast, the low focus-prime congruency (i.e., promotion-*scutum* and prevention-*caligae; M*s = 7.74 and 7.70, *SD*s = 0.93 and 1.02, respectively) conditions did not provoke a higher WTC than the control; *d*s = .13 and .09, respectively. Indeed, taken together, the high focus-prime congruency conditions provoked a WTC that was higher than that provoked by the low focus-prime congruency conditions (*M*s = 8.06 vs. 7.72, *SD*s = 0.67 and 0.97), *d* = .41.

Furthermore, as expected, the overall pattern of results was not found in the WTC with low protective normative appeals: the Waller-Duncan post hoc tests (*p* < .05) indicated that none of the eight conditions that involved either the presentation of the *caligae*-*scutum* prime, the promotion-prevention focus, or a combination of both (3.93 < *M*s < 4.65) significantly differed from the control condition (*M* = 4.28, *SD* = 1.07).

Interestingly, the exclusive use of the *caligae* prime and the prevention focus did increase WTC (*M*s = 8.14 and 8.03, *SD*s = 0.57 and 0.62, respectively), whereas the exclusive use of the *scutum* prime and promotion focus did not (*M*s = 7.74 and 7.95, *SD*s = 0.66 and 0.62, respectively). Regarding this result, it is possible that the person and context combination in which the study was conducted (i.e., university students in a library) had provoked the promotion focus per se, and consequently boosted the influence of the *caligae* prime and reduced the influence of the *scutum* prime. Indeed, the situational elicitation of the prevention focus was effective. Nevertheless, this is a post hoc explanation of a non-hypothesized effect –though consistent with the premises of EMNA– that can be addressed by future research.

Summing up, in line with both the proposed twofold meaning of protection and the motivational relevance accounted by the Regulatory Focus Theory, for the *high protective* normative appeals, the primes of *caligae* and *scutum* were especially effective when preceded by a congruent focus (promotion-*caligae*, prevention-*scutum*). In the same logic, these primes were not effective when their motivational relevance was lowered by an incongruent focus (promotion-*scutum*, prevention-*caligae*).

Therefore, taken together, the results highlight the call for considering separately each of the two meanings, the one related to fostering the performance of the main intended action (*caligae*) and the one related to avoiding the occurrence of physical or psychological damage (*scutum*). Indeed, the objective of the Study 2 is to test whether the willingness to comply with an actually present normative appeal can be predicted by the perceptions of *caligae* and *scutum* taken separately.

## Study 2: Perceiving Caligae and Scutum

According to the EMNA, the more the normative appeal is appraised as fostering the performance of the intended action (*caligae*) and/or avoiding the occurrence of damage (*scutum*) the higher the willingness to comply (WTC) with it. Note that any specific normative appeal can be related more to one meaning than to the other, but their respective association with the WTC can remain separate. In this study, we selected a normative appeal that is pervasive in the academic context (i.e., “To get into a class punctually”) and tested the hypothesized separate power of the perception of *caligae* and the perception of *scutum* to predict the WTC.

### Method

#### Participants and Procedure

A total sample of 513 Spanish (*n* = 95), Chilean (*n* = 151) and Argentinean (*n* = 267) participants (66.3% women, *M*_age_ = 22.19, *SD*_age_ = 3.74) participated in this study. Seven were excluded for not completing the items relevant for this study. The power analyses showed that this sample for a multiple regression study with five predictors allows to localize small size effects (*d* = .05) with power of 80%.

In a general survey conducted to assess several aspects of the academic life, we included six items concerning the objective of this study. Namely, due to its novelty, we decided to measure participants’ perception of *caligae* and *scutum* referred to the normative appeal “One should get into class punctually” through two different approaches. First, approximately half of the sample completed two bipolar items (i.e., “it provokes insecurity vs. it provokes security”, “it stops me vs. it propels me”), while the other half completed two Likert items (i.e., “it facilitates the development of my action”, “it takes cares of myself and my interests”). Regarding the WTC, participants reported “the extent to which they are intended to follow the appeal” and “how probable they will behave according to the appeal”. Participants answered all the items on a 7-point scale (1 = Not at all, 7 = Extremely).

### Results and Discussion

Overall, participants reported a moderately high WTC with the “punctuality” appeal measured either through the “intention” or the “likelihood” items (*M*s = 6.23 and 5.76, *SD*s = 1.12 and 1.34, respectively), as well as a moderately high perception of both *caligae* (*Ms* = 5.22 and 5.93, *SDs* = 1.48 and 1.38 for the bipolar and Liker items, respectively) and *scutum* (*M* = 5.16 and 4.78, *SD* = 1.52 and 1.77 for the bipolar and Liker items, respectively). In line with Study 1, the 2 (gender) × 3 (country) ANOVAs revealed that WTC was higher among women than men [*M*s = 6.11 vs. 5.73, *SD*s = 1.03 and 1.19, respectively, *F*(2, 491) = 9.60, *η*_p_^2^ = .019, *p* = .002]. The country and the interaction effect were not significant, *F*s < 0.65, *p*s > .50, *η*_p_^2^ < .004.

We conducted several stepwise regression analyses to test the relative effectiveness of the perception of *caligae* and the perception of *scutum* in predicting the WTC with the “punctuality” appeal (formed by combining the “intention” and “likelihood” items, *α* = .72). Besides, to control the possible influence of the sociodemographic variables (i.e., gender, country and age), we included them as potential predictors. As shown in Table 2, the general pattern reveals that *caligae* and *scutum* predicted WTC [*R*^2^ = .138, *F*(5, 485) = 16.73, *p* = .001] independently from each other. This pattern remains very similar when the predictors were measured through either bipolar or Likert items, and when the WTC referred only to intention, only to likelihood to fulfill, or both.

**Table 2:** Beta coefficients in the step-wise regression analyses. Predictors: perception of *scutum* and *caligae* in the groups where they were assessed through bipolar (*N* = 257) or Likert (*N* = 248) items, and in the total sample (*N* = 506). Criteria: willingness to comply assessed through intention, likelihood or the combined measure (averaged sum)

	Intention			Likelihood			Combined		
	Bipolar	Likert	Total	Bipolar	Likert	Total	Bipolar	Likert	Total
Scutum	.151^*^	.148^*^	.132^**^	.190^**^	.141*	.183^***^	.219^**^	.161^*^	.179^***^
Caligae	.238^**^	.199^***^	.237^***^	.236^**^	.176^***^	.190^***^	.219^**^	.226^***^	.238^***^
Gender	.155^**^	.045	.091^*^	.014	.149^**^	.084	.032	.169^**^	.098^*^
Country	−.035	.035	.003	.076	−.040	.017	.064	−.044	.013
Age	.029	.032	.030	−.016	−.005	−004	.008	.011	.012
*R* ^2^	.086^***^	.123^***^	.109^***^	.137^***^	.083^***^	.109^***^	.145^***^	.127^***^	.138^***^

*p < .05, **p ≤ .01, ***p ≤ .005

Therefore, the results supported that perceptions of *caligae* and of *scutum* separately predict willingness to comply with a present normative appeal. This was the consistent outcome when using either bipolar or Likert items to measure the predictors, and when the criteria referred to either reported intention or likelihood to fulfill the “punctuality” appeal.

## General Discussion

The results support the twofold meaning of the dimension of protection proposed by the EMNA. Regarding Study 1, when we used the meanings of *scutum* and *caligae* as a prime and combined them with the prevention and promotion focus, willingness to comply with normative appeals followed the pattern coherent with the joint consideration of the EMNA (Oceja et al., 2016) and the Regulatory Focus Theory. Specifically, this willingness significantly increased when there was congruency between the prime and the focus (prevention-*scutum* or promotion-*caligae*) whereas did not when lacking congruency (promotion-*scutum* or prevention-*caligae*). Importantly, this pattern was found only for the willingness to comply with normative appeals that are perceived as protective. These effects cannot be explained in terms of the experimental demands. The randomized between-subjects design, the intermixed presentation of high and low protective normative appeals, and not having previously asking participants regarding their normative appraisal of the appeals assure that those possible experimental demand effects were controlled.

Additionally, the results of Study 2 complement those of Study 1. Now, for a specific and present normative appeal, each of the measured perceptions of *caligae* and *scutum* adds its respective power –not accounted for by the other – to predict willingness to comply. Furthermore, this willingness clearly referred to the reported intention and likelihood of fulfilling the normative appeal.

## Theoretical Connections and Practical Implications

Regarding the Focus Theory of Normative Conduct (FTNC), first, the EMNA agrees on the importance of salience: only a normative appeal that has drawn our attention will be an input of the normative appraisal. Second, the willingness to comply will be high if the normative appeal is perceived as protective (in either the *caligae*, the *scutum* sense, or both) and the action expressed in it is sustained by the perception of what most people do (descriptive norm) or think (prescriptive norm). However, what can we expect if the person perceives the appeal (e.g., You should wear a particular piece of clothing when working in this place) as non-protective though the action is perceived as descriptively and/or prescriptively sustained, or vice versa? (Wesley et al., 2018). The joint consideration of FTNC and EMNA will shed light on this issue.

Regarding the Theory of Normative Activation, the set of values that are personally central and/or made salient by the situation (Verplanken & Holland, 2002) may lead the perceiver to center on the *scutum* or the *caligae* meaning of protection when appraising the normative appeal. Presumably, values related to openness-to-change (i.e., stimulation and self-direction) and to conservation (i.e., tradition, conformity, and security) will increase the prevalence of the *caligae* and the *scutum* meaning, respectively. Therefore, considering personal values that may be prevalent in a particular situation-person combination will enrich the predictions concerning the willingness to comply with specific normative appeals.

Regarding the Relational Model of Authority and the Group Engagement Model, often both the content of a normative appeal and the context in which it is set are ruled by an authority whose decisions and procedures may either concur or collide with the normative appraisal. In these situations, the normative appeal will probably be perceived as formal; however, its perception as low or high protective (a coercive or a legitimate norm, respectively) could depend on the prevalence of the either *caligae* or *scutum* meaning. What can we expect when one authority mostly perceived as fair tries to impose a normative appeal that is mostly perceived as a coercive norm (e.g., You should not use the office equipment for personal matters)?

This question leads to an issue with important theoretical and practical implications. All social systems contain a set of normative appeals that are perceived as formal, but not protective –in the EMNA terms, as *coercive norms*. The results of previous research (Oceja et al., 2016) and this work coincide in showing that these cases provoke a low willingness to comply and, consequently, may eventually incite serious social problems. Two theoretical approaches may be useful to address this issue. First, the Perverse Norm Model (Dols, 1992; Fernández-Dols, 1993) has shown that authorities may ignite effects such as demoralization and corruption within the entire system when declaring that these normative appeals consistently perceived as coercive norms are legally valid (Oceja & Fernández-Dols, 2001; Oceja & Fernández-Dols, 2006). Second, the Anxiety-to-Approach Model (Jonas et al., 2014) comprehensively addresses the cognitive and motivational processes that underlie the people’s reactions to different kind of threats. Therefore, this model is especially useful to understand, anticipate and deal with the complex and compound psychological reaction –that goes beyond a low willingness to comply– provoked by a (formal) normative appeal that it is perceived not just as low protective but as a barrier or a threat. In summary, the development of theoretical concepts and evaluation tools that deepen and advance the ability to anticipate and understand these cases could be of great use in the appropriate management of norms. The EMNA, in conjunction with other models, can collaborate in developing of a theoretically and empirically sound Normology (Morris et al., 2015).

## Limits and Future Direction

The list of 28 normative appeals is incomplete. It was originally conceived by Oceja et al. (2016) to cover as much as possible the perception space formed by the two dimensions of the normative appraisal (formality and protection) and summarized by the four categories (custom, coercive norm, prescription, and legitimate norm). However, future research should include a more comprehensive list of cases selected by content (e.g., environment, health, education), population (e.g., gender, age, psychological characteristics), and context (e.g., work, road, restaurant). Indeed, normative appeals are not usually presented as a package that is evaluated while quietly seated in a library; on the contrary, any specific appeal is perceived in a precise moment and place, and usually while we are in motion (Leoniak & Maj, 2016; Oceja & Berenguer, 2009). We aimed at being close to this reality with the Study 2.

In line with the diversity of the appeals potentially subjected to the normative appraisal proposed by the EMNA, two important questions should be addressed by future studies. First, to what extent the perception of safety (*scutum*) refers exclusively to oneself, or also includes specific others or the society? We propose that it mainly refers to oneself; however, available evidence does not support a conclusive answer. On one hand, perceiving as low protective the normative appeals against internet pirating, photocopying books, and talking on the cell phone in an elevator calls for our proposal. On the other hand, perceiving as high protective those against damaging public property, blocking an ambulance, and using anti-environmental sprays does not discard the alternative. Further research is needed to clearly disentangle the effect of oneself vs. others difference on the *scutum* component.

Second, to what extent the perception of autonomy (*caligae*) depends on the extent to which the action stated in the normative appeal either allows or collides with the individual’s own specific goal in the situation (i.e., the main intended action)? We propose that it does; therefore, we hypothesize that a “be quiet” normative appeal placed in a library will be perceived as low or high protective depending on whether the individual intended to study or meet a friend, respectively. Future research in which this difference is either manipulated or measured will elucidate this question.^1^

The main purpose of the present work was to test whether the dimension of protection –the key component of the normative appraisal proposed by the EMNA– can be divided in two components. We are now in condition to select different concrete normative appeal-situation-person combinations, manipulating and/or measuring the normative appraisal (formality and the *caligae* or the *scutum* meanings of protection) and testing its influence on actual compliance. In other words, we are now better equipped to follow Cialdini’s (2009) advice of conducting field research that addresses this topic in a more organic way.

Taken together, these results call for future research on at least two lines. First, on those factors that may moderate the prevalence of *caligae* or *scutum* right in the moment that the person appraises the normative appeal. These factors are related to the content of the appeal (e.g., an action related to health vs. leisure), the person to whom it is addressed (e.g., a young male student vs. an elderly female), and the context in which it is promulgated (e.g., an office vs. a pub). Second, on the design and examination of measures that may adequately tap the perception of these two components of protection. In this sense, we recently started a line of research to test whether the willingness to comply with a set of norms adopted to address the pandemic COVID-19 (e.g., the confinement, wearing a surgical mask, keeping the social distance, and so on) partially depends on the normative appraisal proposed by the EMNA. That is, does the *scutum*-*caligae* difference shed light on the anticipation and explanation of the change on the degree of adhesion toward these norms over the time?

These future avenues of research could promote new connections among different approaches to the study of social norms, a field typically characterized by fragmentation and isolation. Fortunately, this depiction is changing (for a good example of how to combine theoretical approaches concerning the normative compliance see Jonas et al., 2008; Manning, 2011). This more integrative perspective may lead both scientists and *social script writers* (politicians, judges, lawmakers, organizational authorities, enforcers, etc.) to create normative appeals that nudge *social actors* (citizens, employees, customers, etc.) to comply with them.

## Footnotes

1 We thank a Reviewer of a previous version of this manuscript to have pointed out these two questions
